# Positive Relationship between Total Antioxidant Status and Chemokines Observed in Adults

**DOI:** 10.1155/2014/693680

**Published:** 2014-08-28

**Authors:** Yanli Li, Richard W. Browne, Matthew R. Bonner, Furong Deng, Lili Tian, Lina Mu

**Affiliations:** ^1^Department of Epidemiology and Environmental Health, School of Public Health and Health Professions, State University of New York at Buffalo, Buffalo, NY 14214, USA; ^2^Department of Biotechnical and Clinical Laboratory Sciences, State University of New York at Buffalo, Buffalo, NY 14214, USA; ^3^Department of Environmental Health, Peking University Health Science Center, Beijing 100191, China; ^4^Department of Biostatistics, State University of New York at Buffalo, Buffalo, NY 14214, USA

## Abstract

*Objective*. Human evidence is limited regarding the interaction between oxidative stress biomarkers and chemokines, especially in a population of adults without overt clinical disease. The current study aims to examine the possible relationships of antioxidant and lipid peroxidation markers with several chemokines in adults. *Methods*. We assessed cross-sectional associations of total antioxidant status (TAS) and two lipid peroxidation markers malondialdehyde (MDA) and thiobarbituric acid reactive substances (TBARS) with a suite of serum chemokines, including CXCL-1 (GRO-α), CXCL-8 (IL-8), CXCL-10 (IP-10), CCL-2 (MCP-1), CCL-5 (RANTES), CCL-8 (MCP-2), CCL-11 (Eotaxin-1), and CCL-17 (TARC), among 104 Chinese adults without serious preexisting clinical conditions in Beijing before 2008 Olympics. *Results*. TAS showed significantly positive correlations with MCP-1 (*r* = 0.15751, *P* = 0.0014), MCP-2 (*r* = 0.3721, *P* = 0.0001), Eotaxin-1 (*r* = 0.39598, *P* < 0.0001), and TARC (*r* = 0.27149, *P* = 0.0053). The positive correlations remained unchanged after controlling for age, sex, body mass index, smoking, and alcohol drinking status. No associations were found between any of the chemokines measured in this study and MDA or TBARS. Similar patterns were observed when the analyses were limited to nonsmokers. *Conclusion*. Total antioxidant status is positively associated with several chemokines in this adult population.

## 1. Introduction

Oxidative stress and inflammation are hypothesized to play important roles in a wide variety of diseases, such as cardiovascular diseases, diabetes, cancer, and autoimmune diseases [1–3]. Oxidative stress occurs when antioxidants fail to provide sufficient protection against the overproduced free radicals, and it might lead to oxidative damage to lipids, proteins, and DNA [4–6]. Chronic inflammation is characterized by continuous inflammatory response and tissue destruction [7–11]. A progressive rise of oxidative stress and increased level of proinflammatory mediators appear to be one of the hallmarks of the aging process [3, 12, 13].

Oxidative stress and inflammation pathways are inseparably interconnected [14]. Oxidants activate transcription factor nuclear factor kappa B (NF-*κ* B) that further stimulates production of proinflammatory molecules. Conversely, inflammation causes oxidative stress, because production of free radicals is an inherent property of activated immune cells [14, 15]. The interaction between oxidative stress and inflammation might play an important role in the pathogenesis of a number of human diseases [16–19].

Inflammatory chemokines are a large family of structurally related chemoattractant cytokines that play a pivotal role in orchestrating inflammation [20]. The chemokine system has been demonstrated to be redox regulated in experimental studies [21, 22]. Epidemiological studies reported associations between oxidative stress markers and chemokines among several diseased populations, including patients with acute coronary syndromes, systemic lupus erythematosus and rheumatoid arthritis, preeclampsia, and end-stage renal diseases undergoing hemodialysis [23–26]. However, the potential for the disease process to cause elevated expression of both chemokines and oxidative stress cannot be ruled out. To our knowledge, no human study has been conducted to examine the possible association between chemokines and oxidative stress biomarkers among adults without clinical disease. In the current study, we aim to examine the cross-sectional relationships of serum chemokines with biomarkers of total antioxidant capacity and lipid peroxidation among adults without serious preexisting clinical conditions.

## 2. Materials and Methods

### 2.1. Study Population

A total of 104 participants were recruited from Haidian District, Beijing, in July 2008. Participants were restricted to adults aged from 18 to 70 years, with no previous medical history of cancer, serious immunological diseases, chronic respiratory diseases, cardiovascular diseases, or diabetes. The study population has been described in detail previously [27]. Prior to recruitment, IRB approvals were obtained from the State University of New York at Buffalo and Peking University Health Science Center. Written inform consent was obtained from all study participants.

Trained interviewers administered standardized, structured questionnaires to participants querying information about demographics and lifestyle factors. Current height and weight at the time of interview were measured by trained nurses. Blood samples (5 mL) and first morning urine samples were collected.

### 2.2. Laboratory Analysis of Serum Chemokines and Oxidative Stress Markers

The collected blood samples were immediately transferred to Peking University for processing. Serum and blood clots were separated on the centrifuge and stored at −80°C freezer. All oxidative stress biomarkers were measured at the Department of Biotechnical and Clinical Laboratory Sciences, University at Buffalo. Total antioxidant status rate (TAS) in serum samples was measured using Randox total antioxidant status kit (Randox Laboratories Ltd.) adapted to the COBAS MIRA automated chemistry analyzer (Roche Diagnostic Systems). Biomarkers of oxidative damage to the lipids, including malondialdehyde (MDA) and thiobarbituric acid-reactive substances (TBARS), were measured in urine samples. Total MDA was measured using HPLC according to the procedure described by Giera et al. [28]. TBARS was measured using a colorimetric microplate assay (Oxford Biomedical Research). Both MDA and TBARS were expressed in nmoles/milliliter of MDA equivalents. Urinary creatinine was measured using “Creatinine-a” assay kit (Genzyme Diagnostics) on the ABX PENTRA 400 analyzer (Horiba Instruments Levine, CA, USA). Urinary MDA and TBARS measurements were normalized to the urinary creatinine concentrations to control for variations in urine output. The intra-assay coefficients of variation (CVs) for TAS, MDA, and TBARS were 2.39%, 5.00%, and 3.56%; the interassay coefficients of variation were 7.10%, 9.63%, and 6.30%, respectively.

Chemokines in serum samples were analyzed using the Q-Plex Human Chemokine ELISA-based chemiluminescent assay from Quansys Biosciences. This assay allows the concurrent measurement of chemokines CXCL-1 (GRO-*α*), CXCL-8 (IL-8), CXCL-10 (IP-10), CCL-2 (MCP-1), CCL-5 (RANTES), CCL-8 (MCP-2), CCL-11 (Eotaxin-1), and CCL-17 (TARC). All study samples were analyzed in triplicate in the same plate. The intra-assay CVs using the triplicate samples were all less than 10% (see Supplementary Material 1 available online at http://dx.doi.org/10.1155/2014/693680). External quality control samples collected in healthy volunteers in the USA were repeatedly measured in all the plates to assess the intra-assay and interassay reproducibility. The intra-assay CVs of the external samples were all less than 15%, except for GRO-*α*; the interassay CVs were less than 20%, except for GRO-*α* and IL-8 (Supplementary Material 1). The higher variations for GRO-*α* and IL-8 assays using the external quality control samples might relate to the significantly lower expression levels in the external quality control samples compared to the samples from the study population (Supplementary Material 2).

### 2.3. Statistical Analysis

To describe basic characteristics of the study participants, we calculated mean values and standard deviations for continuous variables and frequencies and percentages for categorical variables. We calculated medians and interquartile ranges (IQRs) for all the chemokines.

Simple Pearson correlation coefficients were calculated to examine the possible relationships between levels of serum chemokines and oxidative stress biomarkers. We used a logarithmic transformation to normalize the distributions for IP-10, Eotaxin-1, TARC, TBARS, and MDA to meet the normality assumption of this model. Partial Pearson correlation coefficients were also calculated by controlling for age, sex, BMI, smoking, and alcohol drinking status.

To examine possible nonlinear relationship and minimize distortion of the Pearson correlation coefficient due to outliers, we further categorized chemokines into tertiles and compared the mean levels (95% confidence intervals) of oxidative stress markers in each tertile of chemokines. *P* values for trend were calculated to assess possible trends of oxidative stress markers across the tertiles of chemokines. We also assessed whether differences in the mean concentrations of oxidative stress markers between the high and low tertiles of chemokines were statistically significant.

All statistical analyses were performed using the SAS 9.3 (SAS Institute, Cary, NC, USA). All statistical tests were two-sided and considered statistically significant at *P* < 0.05.

## 3. Results

Table 1 shows the characteristics of 104 participants (50 males and 54 females) included in this analysis. Average age of the participants was 48.3 years. About 60.6% of study participants were underweight or normal weight, 28.9% were overweight, and 10.6% were obese. Smokers constituted about 35.6% of the participants and about 32.7% were alcohol drinkers. Medians and IQRs of the measured chemokines are also summarized in Table 1.

TAS showed significantly positive correlations with MCP-1 (*r* = 0.15751, *P* = 0.0014), MCP-2 (*r* = 0.3721, *P* = 0.0001), Eotaxin-1 (*r* = 0.39598, *P* < 0.0001), and TARC (*r* = 0.27149, *P* = 0.0053) (Table 2). After controlling for the effects of age, sex, BMI, smoking, and alcohol drinking status, TAS still showed significantly or borderline significantly positive correlations with MCP-1 (*r* = 0.19606, *P* = 0.0518), MCP-2 (*r* = 0.30958, *P* = 0.0018), Eotaxin-1 (*r* = 0.27219, *P* = 0.007), and TARC (*r* = 0.21458, *P* = 0.0329) (Table 2). In the categorized analyses, participants in the higher tertiles of MCP-1, RANTES, MCP-2, Eotaxin-1, and TARC showed significantly or borderline significantly higher levels of TAS (Table 3). Similar patterns were observed when the analyses were limited to nonsmokers (Supplementary Material 3).

TBARS and MDA were not associated with any of the chemokines we measured (Table 2) or in subanalyses restricted to nonsmokers (Supplementary Material 3).

## 4. Discussion

In this cross-sectional study conducted in adults without serious preexisting conditions, we observed significantly or borderline significantly positive correlations between TAS level and chemokines MCP-1, MCP-2, Eotaxin-1, and TARC. TBARS and MDA showed no associations with any chemokines.

Humans have evolved complex antioxidant strategies against prooxidant conditions. The antioxidant defense system has many components, and a deficiency in any of these components can cause a reduction in the overall antioxidant status of an individual. TAS, a measure of overall antioxidant capacity, describes the dynamic equilibrium between different prooxidants and antioxidants in blood [29].

The observed positive correlations between TAS and several chemokines in this study might reflect their interactions in response to xenobiotic insults. Environmental stimuli, such as smoking and air pollution, are known to induce the production of free radicals [30, 31]. Free radicals have been shown to activate redox-sensitive transcriptional factor NF-*κ* B, which may further stimulate the production of chemokines [14, 15]. In response to overproduction of free radicals, TAS might be elevated as a compensatory response to reestablish “redox homeostasis” [32]. In the current study population, we observed higher TAS concentrations among smokers or during period with higher air pollution levels [33]. Therefore, our finding suggested possible biological interactions between chemokines and excessive formation of free radicals in response to xenobiotic insults. One previous study found that TAS was positively correlated with proinflammatory cytokines IL-1*α*, IL-6, and TNF-*α* in bronchoalveolar lavage fluid in lung cancer patients [34]. It implicated that the interactions between chemokines and TAS might further contribute to the pathogenesis of human disease.

Oxidative damages to lipids, proteins, and DNA within cells may occur when the production of free radicals exceeds the antioxidant capacity of the cell [6]. MDA is one of the final products of polyunsaturated fatty acids peroxidation in the cells and it is a widely used lipid peroxidation marker. TBARS is one commonly used marker to measure MDA; however, it lacks specificity [35]. In the current study, we did not observe any associations between chemokines and MDA or TBARS. Contrary to the null associations observed in our current study, positive associations were found between chemokines and lipid peroxidation markers among several diseased populations [23, 24, 26]. Several explanations are possible for the conflicting results. First, antioxidant defense systems for the adults in this study might be activated in response to xenobiotic insults, as we observed higher levels of TAS in smokers or when air pollution was higher. Unlike the diseased populations, the activated antioxidant protection among the current population might be sufficient to maintain the “redox homeostasis,” which may prevent further oxidative damages to the lipids. Therefore, no correlations between chemokines and lipid peroxidation markers were observed in current population. Second, lack of specificity of the lipid peroxidation assays used in this study might be another explanation for the null findings. TBARS assay does not measure MDA exclusively, since it can react to compounds other than MDA. In addition, MDA is also not generated exclusively by breakdown of lipid hydroperoxide [35, 36].

The present study, to our knowledge, is the first epidemiological study that observed positive relationships between chemokines and TAS, a marker of total antioxidant capacity, among individuals without serious preexisting conditions. These findings supported that inflammation and oxidative stress might interact with each other in response to different environmental stimuli and in the pathogenesis of different human diseases. However this study was subjected to certain limitations. First, we only examined a global marker for antioxidant capacity and two lipid peroxidation markers in this study. Future research is warranted to examine the possible correlations between chemokines with individual antioxidants and/or oxidative damage markers to DNA and protein. Second, although TBARS and MDA are the most commonly used measures for lipid peroxidation, the lack of specificity of the two assays might have limited our ability to assess the actual lipid peroxidation status. Third, GRO-*α* and IL-8 demonstrated large interassay CVs using the external quality control samples; however, this might relate to the significantly lower expression levels of GRO-*α* and IL-8 in the external quality control samples compared to our study samples. Lastly, we did not control for uric acid, an important contributor to serum total antioxidant capacity, when evaluating the relationship between TAS and chemokines. Uric acid is a product of purine nucleotide metabolism that has antioxidant [37] as well as prooxidant [38] capacity. In plasma, as much as half of the total antioxidant capacity as measured by total radical scavenging assays like the assay employed in this study has been attributed to uric acid which acts as a scavenger of singlet oxygen, peroxyl radicals, and hydroxyl radicals [38]. Paradoxically in prooxidant environments, particularly intracellular, uric acid has been shown to form free radicals in a variety of radical-forming systems [39]. The prooxidant potential of uric acid does support considerable literature on the epidemiology of uric acid as a risk factor for disease including cardiovascular disease, hypertension, and metabolic syndrome among others [40–48]. The antioxidant-prooxidant paradox has been thoroughly reviewed previously [38]. Here we acknowledge the lack of normalization or adjustment of the plasma TAS levels by uric acid as a limitation of the study design.

## 5. Conclusions

In this cross-sectional study, we observed serum TAS was positively correlated with chemokines MCP-1, MCP-2, Eotaxin-1, and TARC and this implicated possible interaction between chemokines and oxidative stress biomarkers among this group of adults without serious preexisting clinical conditions.

## Supplementary Material

Supplementary Table 1 provides Intra-assay CVs using triplicate study samples as well as the intra- and inter-assay CVs using external quality control samples from healthy volunteers in the US.
Supplementary Table 2 presents the concentrations of chemokines in the current study sample versus the external quality control samples collected from healthy volunteers in the US.
Supplementary Table 3 shows correlation between chemokines and oxidative stress biomarkers among nonsmokers.

## Figures and Tables

**Table 1:** Basic characteristics of study participants.

Variables	Distribution
	Mean (SD)

Age (years)	48.3 (9.1)
BMI (kg/m^2^)	23.7 (3.4)

	*N* (%)

Sex	
Male	50 (48.1)
Female	54 (51.9)
Age groups	
≤40	16 (15.4)
40–50	36 (34.6)
>50	52 (50.0)
BMI categories	
Underweight and normal (<24 kg/m^2^)	63 (60.6)
Overweight (24–28 kg/m^2^)	30 (28.9)
Obesity (≥28 kg/m^2^)	11 (10.6)
Smoking status	
Smokers	37 (35.6)
Nonsmokers	67 (64.4)
Alcohol drinking	
Drinkers	34 (32.7)
Nondrinkers	70 (67.3)

	Median (IQR)

GRO-*α* (pg/mL)	47.20 (25.35, 86.98)
IL-8 (pg/mL)	40.01 (19.63, 92.89)
IP-10 (pg/mL)	75.61 (58.40, 103.89)
MCP-1 (pg/mL)	121.75 (90.56, 163.27)
RANTES (ng/mL)	25.78 (18.89, 32.21)
MCP-2 (pg/mL)	38.45 (27.42, 53.90)
Eotaxin-1 (pg/mL)	146.02 (109.26, 192.29)
TARC (pg/mL)	169.02 (108.40, 243.05)

**Table 2:** Simple and partial Pearson correlation coefficients between chemokines and oxidative stress biomarkers.

	TAS		TBARS		MDA	
	Simple correlation coefficients	Partial correlation coefficients^1^	Simple correlation coefficients	Partial correlation coefficients^1^	Simple correlation coefficients	Partial correlation coefficients^1^
GRO-*α*	−0.01974	0.02379	0.14517	0.17243	0.13731	0.15975
IL-8	−0.13489	−0.14856	−0.09451	−0.10745	−0.00719	0.01839
IP-10	0.05952	0.10126	−0.11125	−0.11266	−0.13101	−0.12015
MCP-1	0.30966∗∗	0.19606	−0.10333	−0.10115	−0.05286	−0.06448
RANTES	0.09351	0.17431	−0.0015	0.04048	−0.02415	0.00592
MCP-2	0.3721∗∗∗	0.30958∗∗	−0.09373	−0.05383	−0.1378	−0.1185
Eotaxin-1	0.39598∗∗∗	0.27219∗∗	−0.03503	−0.03171	0.07175	0.07696
TARC	0.27149∗∗	0.21458∗	0.03133	0.0541	0.03365	0.04368

Note: 1. Adjusted for age, sex, smoking status, BMI, and alcohol drinking. 2. *P < 0.05, **P < 0.01, and ***P < 0.001.

**Table 3:** Concentrations of TAS by tertiles of chemokines.

	TAS level			Crude *P* for trends	Adjusted *P* for trends	Crude *P* for high versus low tertile	Adjusted *P* for high versus low tertile
	Low tertile	Middle tertile	High tertile				
GRO-*α*	0.94 (0.81, 1.06)	1.03 (0.92, 1.14)	0.94 (0.85, 1.04)	0.9753	0.9454	0.9633	0.8587
IL-8	0.99 (0.89, 1.09)	1.06 (0.94, 1.17)	0.87 (0.77, 0.97)	0.1070	0.1203	0.1022	0.1105
IP-10	0.92 (0.81, 1.03)	1.02 (0.91, 1.13)	0.98 (0.87, 1.08)	0.4363	0.1661	0.4357	0.1658
MCP-1	0.87 (0.78, 0.97)	0.95 (0.83, 1.06)	1.10 (1.00, 1.20)	**0.0021**	0.1772	**0.0022**	0.1808
RANTES	0.89 (0.78, 1.00)	1.03 (0.94, 1.13)	0.98 (0.86, 1.10)	0.2159	**0.0138**	0.1721	**0.0077**
MCP-2	0.83 (0.73, 0.94)	0.98 (0.89, 1.08)	1.10 (1.00, 1.20)	**0.0003**	**0.0058**	**0.0004**	**0.0061**
Eotaxin-1	0.81 (0.72, 0.89)	0.99 (0.90, 1.08)	1.12 (1.00, 1.24)	**<0.0001**	**0.0037**	**<0.0001**	**0.0039**
TARC	0.90 (0.80, 1.00)	0.93 (0.83, 1.03)	1.08 (0.97, 1.20)	**0.0133**	0.1038	**0.0133**	0.1091

## Abbreviations

TAS: Total antioxidant status
MDA: Malondialdehyde
TBARS: Thiobarbituric acid-reactive substances
95% CI: 95% confidence interval
IQR: Interquartile ranges
CV: Coefficients of variation
ICC: Intraclass correlation coefficient
BMI: Body mass index
NF-*κ* B: Nuclear factor kappa B.
