# Supplementary material for: Positive Relationship between Total Antioxidant Status and Chemokines Observed in Adults
Source: Oxid Med Cell Longev. 2014 Aug 28;2014:693680. doi: 10.1155/2014/693680 (PMC4164799; doi:10.1155/2014/693680)
Supplement: Supplementary file 1 — Supplementary Table 1 provides Intra-assay CVs using triplicate study samples as well as the intra- and inter-assay CVs using external quality control samples from healthy volunteers in the US. Supplementary Table 2 presents the concentrations of chemokines in the current study sample versus the external quality control samples collected from healthy volunteers in the US. Supplementary Table 3 shows correlation between chemokines and oxidative stress biomarkers among nonsmokers. [file 693680.f1.zip › table1,2.docx]

Supplementary Material 1. Intra- and Inter-Assay Reproducibility of Chemokines

|  | Intra-assay CVs using triplicate study samples | Intra-assay CVs using external QC samples | Inter-assay CVs using external QC samples |
| --- | --- | --- | --- |
| GRO-α | 7.20% | 39.62% | 61.82% |
| IL-8 | 5.86% | 10.19% | 46.75% |
| IP-10 | 4.73% | 9.22% | 16.19% |
| MCP-1 | 6.29% | 11.63% | 13.46% |
| RANTES | 6.49% | 15.18% | 15.77% |
| MCP-2 | 4.01% | 6.59% | 18.55% |
| Eotaxin-1 | 4.50% | 8.03% | 18.72% |
| TARC | 5.01% | 7.56% | 14.32% |

**Supplementary Material 2. Concentrations of Chemokines in the Study Samples and External Quality Control Samples**

| Chemokines | Median (IQR) in Study Samples | Median (IQR) in External Quality Control Samples |
| --- | --- | --- |
| GROa | 49.21 (26.22, 89.60) | 6.97 (0.90, 16.32) |
| IL-8 | 40.31 (19.20, 101.67) | 10.03 (3.83, 11.22) |
| Eotaxin-1 | 148.29 (110.24, 194.64) | 184.65 (163.70, 209.91) |
| IP-10 | 75.83 (58.08, 103.92) | 43.12 (38.67, 46.78) |
| MCP-1 | 121.65 (90.41, 161.95) | 180.85 (161.57, 195.05) |
| MCP-2 | 38.36 (27.53, 53.68) | 35.13 (31.73, 38.41) |
| RANTES | 25.89 (18.78, 32.67) | 22.09 (20.08, 23.91) |
| TARC | 169.37 (107.81, 242.01) | 299.39 (268.55, 347.87) |

1. Units of all chemokines, except for RANTES, presented in this table are pg/ml. Unit of RANTES is presented in ng/ml.

# References
