# Supplementary material for: Positive Relationship between Total Antioxidant Status and Chemokines Observed in Adults
Source: Oxid Med Cell Longev. 2014 Aug 28;2014:693680. doi: 10.1155/2014/693680 (PMC4164799; doi:10.1155/2014/693680)
Supplement: Supplementary file 1 — Supplementary Table 1 provides Intra-assay CVs using triplicate study samples as well as the intra- and inter-assay CVs using external quality control samples from healthy volunteers in the US. Supplementary Table 2 presents the concentrations of chemokines in the current study sample versus the external quality control samples collected from healthy volunteers in the US. Supplementary Table 3 shows correlation between chemokines and oxidative stress biomarkers among nonsmokers. [file 693680.f1.zip › table3.docx]

**Supplementary material 3. Simple and Partial Pearson Correlation Coefficients between Chemokines and Oxidative Stress Biomarkers in Baseline (Nonsmokers Only)**

|  | TAS | |  | TBARS | |  | MDA | |
| --- | --- | --- | --- | --- | --- | --- | --- | --- |
|  | Simple Correlation Coefficients | Partial Correlation Coefficients^1^ |  | Simple Correlation Coefficients | Partial Correlation Coefficients^1^ |  | Simple Correlation Coefficients | Partial Correlation Coefficients^1^ |
| GRO-α | 0.05939 | 0.00431 |  | 0.16254 | 0.14367 |  | 0.22125 | 0.2086 |
| IL-8 | -0.0733 | -0.10438 |  | -0.10633 | -0.09935 |  | 0.04874 | 0.05193 |
| IP-10 | 0.02124 | -0.00306 |  | -0.13432 | -0.14398 |  | -0.10187 | -0.0905 |
| MCP-1 | 0.37076** | 0.27562* |  | -0.18022 | -0.21391 |  | -0.12484 | -0.17303 |
| RANTES | 0.13794 | 0.17577 |  | -0.0591 | -0.01967 |  | -0.03766 | -0.0091 |
| MCP-2 | 0.31966** | 0.25372* |  | -0.13149 | -0.13198 |  | -0.15689 | -0.15785 |
| Eotaxin-1 | 0.41149*** | 0.28145* |  | -0.01324 | -0.04902 |  | 0.11964 | 0.09532 |
| TARC | 0.19748 | 0.14613 |  | -0.00704 | 0.00639 |  | 0.04678 | 0.05346 |

Note: 1. Adjusted for age, sex, smoking status, BMI and alcohol drinking.

2. * p<0.05, ** p<0.01, *** p<0.001
